# From “a Fair Game” to “a Form of Covert Research”: Research Ethics Committee Members’ Differing Notions of Consent and Potential Risk to Participants Within Social Media Research

**DOI:** 10.1177/1556264617751510

**Published:** 2018-01-19

**Authors:** R. A. Hibbin, G. Samuel, G. E. Derrick

**Affiliations:** 1 Lancaster University, Lancashire, UK; 2 King’s College London, Strand, UK

**Keywords:** social media, ethics, research ethics committee, privacy, consent

## Abstract

Social media (SM) research presents new challenges for research ethics committees (RECs) who must balance familiar ethical principles with new notions of public availability. This article qualitatively examines how U.K. REC members view this balance in terms of risk and consent. While it found significant variance overall, there were discernible experience-based trends. REC members with less experience of reviewing SM held inflexible notions of consent and risk that could be categorized as either relying on traditional notions of requiring direct consent, or viewing publicly available data as “fair game.” More experienced REC members took a more nuanced approach to data use and consent. We conclude that the more nuanced approach should be best practice during ethical review of SM research.

## Introduction

Social media (SM) provides a new methodological research tool allowing researchers to draw on data from websites such as Facebook, Twitter, and YouTube, as well as password-protected and non–password-protected chatrooms and forums (Kaplan & Haenlein, 2010). The benefits of this research center on the huge repository of easily accessible, seemingly public data, available at low cost. However, different views on the publicly available nature of SM platforms and the perceived privacy of SM users present an ethically gray area for research ethics committees (RECs) charged with ensuring that research using SM data is conducted responsibly and ethically.^1^ Ordinarily, researchers’ responsibilities to their research participants are outlined in a range of disciplinary codes of conduct (BERA: British Psychological Society [BPS], 2010; Jones, 2011), and in the off-line context, these responsibilities have clear boundaries that are familiar to researchers and RECs alike (Hedgecoe, 2016). However, in the context of SM research, new challenges that obscure the “fundamental rights of human dignity, autonomy, protection, safety, maximization of benefits and minimization of harms” (Markham & Buchanan, 2012, p. 4) are presented that render familiar ethical principles considerably less so. As such, when ostensibly publicly available data are collected without SM users’ awareness and/or permission (as can be the case with research involving powerful or marginalized groups; Hammersley, 2009), privacy violations based on improper access that have been identified as a concern by certain authors (Zimmer, 2010) can take place that would have been unlikely to occur through research lacking an SM component.

The issue of consent is particularly problematic in the case of SM research. First, consent may not always be possible or practical. Second, while consent is an ethical and legal requirement for any human participant research (HPR; World Medical Association, 2001), a number of scholars reject that SM data fulfill the definition of HPR, with this remaining an ongoing live issue in the literature (Henderson, Johnson, & Auld, 2013; Hutton & Henderson, 2015; Keim-Malpass, Albrecht, Steeves, & Danhauer, 2013; Metcalf & Crawford, 2016). Third, the static, compulsory consent that takes place at the beginning of the research process has been questioned by some SM researchers, who prefer the use of changeable constructs of consent based on the perceived risk to SM users as research participants (Hutton & Henderson, 2015). And finally, some scholars argue that terms and conditions consent—where SM users agree to a platform’s data use policy prior to creating an account—are problematic in acting as a proxy for more rigorous attempts to seek permission from SM users for research (Morrison, McMillan, & Chalmers, 2014). This latter issue has come to the fore in a number of high-profile cases, including a joint Facebook–Cornell University study in 2014 where empirical research on behavior and mood in response to variations in emotionally charged content viewed by users was published without the Facebook users’ knowledge (Kramer et al., 2014). As suggested by Zimmer (2010), when data are “considered freely accessible for collection and research, regardless of what the subject might have intended or desired” (p. 322), privacy violations through the improper access are a potential concern. This issue is only likely to grow alongside the rising profile of big data research and the lack of sufficient regulation regarding ethical best practice for SM research (Vayena et al., 2016).

Such challenges have not been entirely resolved by the guidelines that currently exist to steer best practice (e.g., see Ess, 2002; Markham & Buchanan, 2012), and the uncertainty surrounding ethical decision making for SM research is further reinforced by the bottom-up, researcher-led approach that has been advocated by the Association of Internet Research (AoIR: Ess, 2002) and others (Markham & Buchanan, 2012).

The complexity involved with the interplay between privacy, user awareness, and consent as well as the lack of a standardized approach to SM research means there is little to guide RECs in their decision making. Indeed, preliminary evidence from the US (Buchanan & Hvizdak, 2009), and also in the form of a survey from a U.K. university (Carter et al., 2016) suggests that RECs (and their institutional review board counterparts) are struggling to adapt to these unique challenges during their review of such research (Boyd & Crawford, 2011; Henderson et al., 2013).

This article builds on this research and provides a deeper exploration of how REC members from a number of different U.K. institutions balance the benefits of SM research with the potential harm to SM users during their review of such research. In particular, this article examines how the notions of risk and consent in SM research are considered by REC members. Below we provide a brief review of the differing notions of consent that need to be considered for SM research, thereby highlighting the potential challenges facing REC members assessing research ethics applications involving SM data. We then present our study, which involved interviews with 19 REC members. Our findings explore the level of SM experience of our REC member interviewees and how this influences their conceptualization of consent and risk of SM ethics applications. Our discussion will contextualize our findings within the current developments of SM research and provide recommendations of how to implement a broader range of protections for SM users as participants within research. We note that as an in-depth, small-scale pilot study, it is not possible to generalize the findings reported here across all contexts. However, the broad conclusions that we draw are designed to anchor further explorations of the ethics of SM research in data-rich preliminary work.

## Literature Review

There is a significant variation of opinion in terms of when consent is needed in SM research due to a lack of consensus about a whole range of issues. Some researchers argue that consent is needed as SM data represent HPR; others reject this proposition, rather viewing SM data as “published text” that precludes the permission of the SM user for data use. Beliefs about consent are also influenced by whether researchers view SM data as residing within the public domain (and therefore freely available for any researcher to use) or as private data where SM user expectations of any “perceived privacy” about their data need to be considered during the research process (Markham & Buchanan, 2012; Moreno, Goniu, Moreno, & Diekema, 2013; Samuel, 2017). The type of research being conducted (quantitative versus qualitative data sets) and the discipline within which it is occurring can also play an important role in how researchers view the necessity to ask SM users to source their data (Paechter, 2013).

This lack of consensus results in RECs struggling to know how best to apply familiar ethical principles to SM research that blurs boundaries and provides a mix of different ideas about research (Boyd & Crawford, 2011; Buchanan & Hvizdak, 2009; Carter et al., 2016; Henderson et al., 2013). Indeed, while the BPS (2007) has identified the (a) level of identifiability and (b) level of observation as two key dimensions affecting ethical consideration, within this framework, while consent is something that is required for identifiable and recruited participants, when subjects are not identifiable and/or unaware of being researched, consent becomes an uncertain requirement (BPS, 2007).

Without clear consensus about under what conditions consent for SM data is required, it is uncertain whether the use of SM data should be treated as being publicly available for research purposes. It is similarly uncertain what the risks to participants may be when consent is not considered to be something that is required. In these situations, researchers typically rely on de-identifying their SM data as a standard requirement. However, recent research has found that even large-scale aggregated data can be easily re-identified upon release (Gymrek, McGuire, Golan, Halperin, & Erlich, 2013; Homer et al., 2008; Narayanan & Felten, 2014), calling into question the extent to which de-identification can be considered a sufficient protection when dealing with SM data.

Furthermore, there is a lack of clarity in terms of the extent to which SM users are cognizant of the uses of their data, with only a few recent studies examining user perceptions of SM research. Such studies have found that users want more privacy regardless of the public nature of data (Williams, Burnap, & Sloan, 2017), and overall, they forward a view of research utilizing SM data that upholds the ethical principles of consent, anonymity and avoiding undue harm to participants (Beringer et al., 2014). Beringer et al. (2014) reported that users positioned the need to improve the representativeness of findings and understand the privacy risks of platforms as being paramount to protection and trust of participants. Transparency in reporting results, while protecting the identity and reputation of participants, was central in maintaining their trust and continued support of research.

In addition, recent studies have documented a “privacy paradox” (Barnes, 2006) where SM users’ self-declared desire for more privacy online conflicts with their actual behavior disclosing personal information on websites and mobile apps. It has been suggested that this contradictory behavior may be the result of a number of factors ranging from a lack of understanding and knowledge of risk and privacy-protective behaviors (Acquisti & Gross, 2006; Hargittai & Litt, 2013; Park, 2013), to apathy and learned helplessness in the face of the perceived privacy that SM users have not actually lost, but rather they never had in the first place (Hargittai & Marwick, 2016). In addition, the need to accept consent on SM platforms as a “take it or leave it” offer (Custers, 2016, 2013) is likely to further exacerbate such feelings of apathy and learnt helplessness. Some scholars therefore suggest that consent processes within SM are inadequate and do not address the cognitive problems undermining privacy self-management in terms of SM users making decisions about privacy only selectively and on an often uninformed basis (Custers, 2013; Solove, 2012).

In response to the complexities of ethical decision making during SM research, Vayena et al. (2016) have suggested that “the current regulatory framework emphasizes practices, such as obtaining informed consent and balancing the benefits of research against the risks of participation, that are out of place in non-clinical research” (p.426). They and others argue that while consent can happen in a number of different ways (ranging from lengthy consent forms to less onerous approaches such as opt-out or terms and conditions consent), it is unclear which is the best way for SM research relative to the practical and pragmatic difficulties presented in seeking consent from subjects that are virtually and sometimes geographically distant from researchers. In addition, they also argue consent as a cornerstone of HPR is often inadequate due to consent forms that are “lengthy, complex and difficult to understand”; disclosures that “often do not inform subjects of all potential data uses and the harms that could result from misuse”; and limited opportunities for subjects to “withhold, revoke, or modify consent” (Vayena et al., 2016, p. 432). As a consequence, they say, obtaining consent for SM research can amount to inadequate approaches to subject permission as per the norms of traditional approaches to participant consent and potential risk.

Others similarly argue that it is difficult to formulate simple general rules in SM research that rather needs to be an “inductive process” (Henderson et al., 2013, p. 5; McKee & Porter, 2008). Indeed, it is suggested that there is “no one-size-fits-all approach to consent that achieves optimal results,” and that while low burden “secured consent” where permission is given at a single point in time may be sufficient in some contexts, more sustained approaches where permission is continually probed may be required for other participants in different research contexts (Hutton & Henderson, 2015, p. 186). In addition, while ethical norms also include SM users’ rights not to be researched, this is not always practical or realistic, particularly in light of the seemingly public availability of SM data and arguments that users should be cognizant and aware. Vulnerable populations provide an additional level of complexity to this argument, where participants’ understanding of the publicness of ostensibly private spaces online may be compromised (Henderson et al., 2013).

Vayena et al. (2016) have therefore called for a new ethical framework for research utilizing SM, particularly in relation to big data, which broadens the concepts of protection and consent, and suggests researchers should be selecting from the “wide range of procedural, economic, legal, educational, and technical protections that are available” (p. 437). In other words, other protections beyond direct consent and de-identification need to be considered to ensure that participants are adequately protected in the context of SM research. As with any new development, standards will not emerge overnight. In the meantime, REC members need to balance these tensions regarding risk and consent when considering ethics applications for research proposals that include an SM component. This article will go some way toward illuminating the considerations that RECs are faced with, when reviewing SM research applications.

## Method

### Recruitment

Websites of the 20 most research-intensive U.K. universities (as determined by the U.K. Research Excellence Framework, 2014 ^1^) were searched to identify relevant contact details of Chairs and/or members of their university-level and departmental/faculty-level RECs. Specific interest was placed in identifying those RECs who were responsible for reviewing research which adopted SM approaches in health. As such, RECs solely within physical or life sciences were excluded.

Where contact details were identified, emails requesting participation in the project were sent to the Chair of each REC, as well as to REC members whose areas of expertise were health and social science (i.e., excluding REC members with expertise solely in clinical, life, or physical sciences, as determined by their webpages). Participant information sheets were attached to all requesting emails. Two follow-up emails were sent to nonresponders.

In total, 63 individuals were contacted to request participation in the project. Nineteen individuals responded and interviews were arranged. Participants represented 13 U.K. institutions across 18 different university-level or faculty/department-level (social sciences, humanities, medical sciences, psychology, or health sciences) RECs. They included nine REC Chairs, one deputy Chair, and nine REC members. Ten participants sat on a university-level REC, and 13 participants sat on a departmental/faculty-level REC (four participants sat on both). On questioning about their experience with the ethical review of research which uses SM data (health or otherwise), four stated no such experience (though one of these interviewees had some experience using SM data within their own research); eight noted limited experience; and seven stated having experience.

### Interviews

Interviews were conducted by GS either face-to-face, over the telephone, or via skype. Interviews lasted between 40 and 60 min and were digitally recorded. The interview schedule was broad, asking participants about their own experience of using SM data for research, as well as their general use of SM more generally in their professional or personal life. The interview schedule also explored interviewee’s views about the ethical issues surrounding the use of SM data for research (in particular to health research); their views about whether such research should require ethical approval, and knowledge about the policies at their own institution in relation to this; their experiences of reviewing such research in a REC capacity and their decision making in relation to this; any guidelines, training, or literature they had used to aid their decision making in this area; and, for those with no experience in this area of ethical review, how interviewees thought they would make decisions about this research in their capacity as a REC Chair/member.

### Analysis

Analysis of interview data was approached using inductive reasoning employing the inductive approach of grounded theory (Charmaz, 2006; Strauss, 1987). The analysis (or coding) of data was based on two interlinked rounds: overview analysis and detailed analysis (Strauss, 1987). Overview analysis consisted of memo-making and broad coding. Extensive memo-making was used by the interviewer directly after each interview. Broad coding proceeded by scanning the interview transcripts for relevant ideas and themes. This phase was conducted in duplicate by first and second authors, before one theme per author was selected for detailed analysis on the basis of extensive team discussion.

### SM Experience of Participants

Participants’ experience was classified into three levels: (A) experienced; (B) limited experience; and (C) inexperienced. Levels of experience were determined during the interviews with the participants self-categorizing and/or making direct reference to their own perceived level of experience. Participants were noted to have direct experience if they had conducted and/or reviewed more than one SM study (columns A-B in Table 1).

**Table 1.** Participants Self-Declared Levels of SM Experience.

A	B	C	D	E
Experienced conducting SM research	Experienced reviewing SM research applications	Limited experience conducting SM research	Limited experience reviewing SM research	Complete inexperience
4	6	8	8	2

Participants were categorized as having limited experience if their practice of conducting and reviewing SM studies had been indirect (through other collaborators or students), or limited to a single study. In addition, if participants owned and used an SM account either personally or professionally, this was also counted as indirect experience (columns C-D in Table 1).

On the contrary, inexperience consisted of having no experience at all with conducting or reviewing SM studies, and not owning an SM account for personal or professional use (column E in Table 1).

For the remainder of the article, when we talk about “experienced” REC members we are referring to those occupying categories A-B; when we talk about REC members having “limited experience” we are referring to those within categories C-D; and when we talk about the minority of participants labeled as entirely “nonexperienced,” we are referring to those within category E (see Note 2).

## Results

### Do No Harm in Practice: Context, User Awareness, and De-Identification

REC members centered their ethical questioning of SM research applications on the principle of “do no harm.” This involved engaging with “a whole trench of really familiar ethics questions”; and “standard worries around confidentiality and consent, and whether privacy has been respected” (REC 14). In practice, “do no harm” generally required all research participants to be adequately informed about the project, to have consented to the research they are participating in, and to have had their identities protected. These traditional ethical norms (Vayena et al., 2016) were relatively unproblematic when a nonexceptionalist approach was taken (Gelinas et al., 2017) and SM research proposals had already written the need for SM user consent into the research methodology. However, all REC members were aware that when explicit consent had not been sought from SM users (as is often the case for SM research; see Henderson et al., 2013), applying the “do no harm” principle was complicated by the presence of SM data being seemingly freely and publicly available. In these instances, REC members had a well-developed awareness that SM users could not be assumed to be aware their data were being used for research purposes (Henderson et al., 2013; McKee & Porter, 2008; Moreno et al., 2013). This idea of SM user “perceived privacy” raised the question of whether any research proposal that did not ask for SM user consent was ethically problematic.

To address this, REC members drew on other strategies to help inform their ethical judgments when reviewing SM research. These included looking at the context of the research (i.e., the specific topic under investigation) and “what actually are the questions that are being asked from the data that you’re collecting on people” (REC 7). More “benign” subject matter was generally required as requiring less scrutiny in terms of consent, because of a perceived decrease in risk to SM participants:

I still don’t like the idea [of not asking for consent], you’ve got something on me that I didn’t consent to, you can sort of argue on principle, that’s not a good thing to do, but it’s still benign so it doesn’t really matter [for example if the topic is shoe preferences]. But it becomes more of an issue when particularly the context that you—the theme of your work which is health. (REC 7)

This was contrasted with more sensitive research topics (health-related, pregnancy blogs, or radical religious views expressed on Twitter), where there was more chance of harm being done to the research participant through the collection of SM data. Beyond the principle of “do no harm” that was of central importance to all REC members, varying levels of personal and professional experience shaped the way that interviewees viewed risk and consent in the context of SM research. We go on to explore these differing views in relation to REC member experience of SM research, below.

### Inexperienced REC Member Views: The Perceived Need for Consent

The majority of REC members with limited experience of SM research and/or review who perceived the need for consent for all SM research spoke about falling back on strong traditional notions of ethical behavior around consent,^3^ and approaching ethical review as they would for non-SM research, “look[ing] at it in terms of the usual principles apply” (REC 15). These REC members felt uncomfortable allowing researchers to use SM data without consent, “even if it’s for the good of society” because users would be “aggrieved” (REC 7). Indeed, one interviewee likened their concerns to people speaking privately in a public staff canteen and their conversation being publicized out of context. Another interviewee explained how, even on public (non–password-protected) SM sites, researchers need to be sensitive to their use of SM data, and realize that there are “people behind the data” who need to be “consented,” and made aware. In these instances, the “general principle” taken by less experienced REC members was that researchers “should attempt to inform and ask consent” (REC 3).

Having said this, there was also a realization by these particular interviewees that using this nonexceptionalist approach (Gelinas et al., 2017), that is, analogizing SM research to its non-SM counterpart, did not always work in practice, because of the fast-paced and ever changing nature of SM platforms and associated research methodologies. For some particularly inexperienced RECs, there was a degree of anxiety attached to the dynamic and changeable nature of SM as a technology, where uncertainty and concerns about “known unknowns” (REC 12) were viewed as something to be worried about in a somewhat reactionary way. It is likely that such uncertainty around not knowing “the potential harms until they happen” resulted in these REC members taking an even more cautious stance in relation to the perceived need for consent:

The way that it’s [SM] being used you would never know what’s next. Because when Facebook came in, there was also Myspace and other platforms were available. And people try to understand how to engage with those kinds of platforms and what were the ethics concerned when you were doing that. And then some other social media comes the next day, and it’s a completely different way of dealing with it and you don’t know. So . . . you don’t know yet what they are or what are the potential harms until harm happens . . . (REC 1)

Compounding all the difficulties and uncertainty expressed by less experienced REC members in relation to reviewing studies that included an SM component were concerns relating to the lack of clear “guidelines on how you deal with these issues” that can make it “really hard” (REC 18) in the absence of a reliable normative framework. In spite of less experienced REC members struggling with the idea of using a nonexceptionalist approach in a changing research field, they tended to forward a view of SM research that upheld old notions of the need to gain consent regardless of the practical difficulties obtaining it. This was because these interviewees were guided by a deep sense of responsibility to protect SM users—a view that was also shared by the REC members who were more experienced in both SM research and also review (*n* = 10):

Some people will say okay, it’s in the public domain so I can use it as I want. Actually as researchers . . . [we can] never use anything just as we want. We always have to look at the protection of participants, potential harms, whether or not it’s us causing it or them causing it in a way . . . And if we’re manipulating that as researchers, we have certain ethical requirements that we need to adhere to. (REC Member 4)

Here, it was vital that SM user expectations (user awareness) were considered as part of best practice ethical decision making, whereby thinking about “the likely expectations of the people” (REC 11) was paramount. For some interviewees, failing to take these user expectations into account meant that SM research could be compared to “covert research,” that did not apply the traditional ethical norms of consent and awareness of being researched:

There’s something about social media that people are less informed about how their data might be used . . . it feels more of a form of covert research in some ways because people are carrying out their research when other people who use social media may not be aware of it. (REC 19)

Such a view was strengthened in situations where SM data were sourced from password-protected sites. Here, interviewees placed even more responsibility upon themselves to protect SM users and to seek permission to use SM data, or at least make SM users aware that their data could be used for research purposes. In these instances, consent was viewed as being required by the majority of interviewees regardless of experience (*n* = 17):

We certainly discussed the nature of the platform and distinction between public facing things like twitter and closed chat groups, very much an ethics committee would be mindful of certainly because I think that those lead to a difference in how we would view the privacy element of that. (REC 16)

Overall then, for less experienced REC members, consent was seen as a compulsory element for any SM research, rather than something that could be applied more selectively depending on the perceived risk to SM users.

### Inexperienced REC Member Views: Fair Game

The alternative view taken by a minority of REC members with limited experience of conducting and/or reviewing SM research was that most SM users were aware that their data could be used for research purposes:

I think people are cognizant of the danger that they’re using that kind of technology . . . that they might put up or post or upload to those sites, the way those sites can be used, co-opted by different people for different purposes . . . (REC 8)

These REC members (*n* = 7) forwarded an almost laissez-faire view and saw SM data as “fair game” for research purposes where “if you’re prepared to put the stuff in the public domain, then it’s in the public domain” (REC 2). This view was particularly prominent for those with very limited experience of SM research that amounted to indirect experience of research and review of a single SM-based study.

. . . if there is something that I can just access to somebody’s data just by googling it, and then I don’t need—or a researcher doesn’t need consent from the participant because already it’s readily available. Why bother? (REC 1)

Alongside this “fair game” notion of SM data, there was an expectation that users *should* be aware of the public nature of online platforms, and it was not for REC members to be responsible for those who are not. This attitude was especially true if SM users were posting on a public (non–password-protected) site, such as Twitter:

The question is, “are they responsible for not knowing or should someone be telling them?” And I suppose I tend to think that people have a certain responsibility for themselves . . . we don’t remain children throughout our lives and the stuff that people put on social media I think sometimes is very self-destructive and stupid but they can’t really say they didn’t know it’s public information. And if you don’t want stuff to be made public then don’t make it public. (REC 10)

The responsibility of the REC member was therefore generally seen as protecting against harm by solely ensuring that the potential for identifying SM users from their data was minimized within both large-scale quantitative studies and small-scale qualitative studies. The use of SM data without consent was therefore perceived as ethically unproblematic, especially in public forums, so long as users remained de-identified:

. . . what are the risks to the individual if this data is collected on them that they haven’t consented to. Actually the risks are minimal, because, you can’t trace it back to who they are . . . (REC 7)

Moreover, for some “pretty liberal” (REC 10) interviewees, this risk-based approach to ethical review applied to research proposals sourcing SM data from a private (password protected) forum:

If you said, “look it’s crucial we understand what people are discussing in a particular chat room and we’re only interested in what they say, not who they are and the interactions that they’re having and the way in which they interact” I suppose I would err on the side of being pretty liberal for that as long as the investigator was simply an observer or something. . . . I mean it’s not like they’re breaking into someone’s house and overhearing their conversation. They’re actually free to join, they’re free to listen. (REC 10)

Here then, REC members with limited or no experience conducting and/or reviewing studies that included an SM component tended to forward a view of SM research that emphasized a fair game view that resulted in the perception of a reduced need for consent overall. It is worth noting however that less experienced REC members sometimes held both traditional notions of the need to consent alongside fair game views of publicly available data (e.g., REC 7). This highlights the complexity and uncertainty of ethical decision making in relation to risk and consent in the online context.

### Experienced REC Member Views: The Nuanced Middle Ground

For the more experienced REC members with direct professional experience of conducting SM research (*n* = 4), the fair game argument of publicly available data was considered a “naïve view” (REC 16) and a “dangerous thought” (REC 14), which overlooked the complexities of user awareness and the responsibility of researchers to do no harm as a minimum standard. However, the need to always seek consent was also problematic for these REC members due to pragmatic considerations about study design where seeking consent was not always desirable or possible. Rather, more nuanced views that were simultaneously less cautious in relation to consent, while being more mindful of participant protections that could be practically applied (as opposed to the fair game argument that saw SM data as being entirely ‘up for grabs’ with little or no consideration of practical solutions to protecting participants) tended to be expressed by these REC members.

While a variety of views were expressed by more experienced REC members, those who upheld a strong sense of responsibility in terms of protecting participants explained that while in an ideal world they would prefer researchers to ask for SM user consent directly, they understood the impracticalities presented by SM research in relation to this aim. Methodology was an important factor in determining when it was appropriate to resort to notions of negotiated consent from SM users. This negotiated level of consent was perceived appropriate for studies using large-scale data sets because they presented a much lower level of risk:

If we’re data mining 10,000 Twitter feeds . . . I don’t think it’s really necessary because we’re not identifying anybody, we are aggregating those data, we’re not disaggregating them. So, again, it’s the actual research practice that’s involved . . . no, we don’t need to really—asking a person’s consent . . . it’s not even their data that we’re accessing, it’s what their data does which is contribute to a spike in the use of a particular hashtag . . . it’s not even the content of what they said. (REC 13)

Alongside this, interviewees were “realistic” about the practical difficulties of gaining consent for SM research. For example, instances in which researchers are harvesting “millions of tweets” (REC 17) consent was not seen as being either practical or possible. Similarly, where SM users are chatting on an anonymous chatroom or had left the discussion, gaining consent was not seen as something researchers could easily achieve:

But the informed consent process obviously, that will be very, very difficult to do. So you have to be realistic as well and I think, well, how would you email millions of people and would then do an opt out or opt in process or what—so I think, in that case, in the past, we have said, well, okay, as long as nobody is harmed by this research in anyway and it’s not against any terms and conditions then, it’s probably okay. (REC 17)

In addition, dilemmas about altering user behavior through attempting to obtain consent from participants were raised by interviewees:

And so the question arose whether that method of data collection met criteria for informed consent . . . they feared that if they announced themselves on this chatroom site that people wouldn’t be candid . . . so I thought, it’s quite a dilemma. (REC 5)

REC member 17 explained how choosing to not ask for consent in these instances was not necessarily ethically problematic, so long as there was no potential for the SM users to be harmed during the research process. Indeed, when “secured consent” (Hutton & Henderson, 2015) was unavailable at the point of data collection due to the need for unobtrusive researcher presence that did not “destroy the research” (REC 11), or impracticalities obtaining consent directly, best practice was framed by interviewees as including safeguards that could provide information to participants on the research being conducted; disclose researcher presence within forums; and enable retrospective consent and opt-out processes for research participants. In addition, negotiated consent processes were positioned as safeguards that could produce varying levels of research integrity and participant protection. For example, asking the moderator of a forum for consent was viewed as being more ethically rigorous than a terms and conditions disclosure that represents little more than a superficial box-ticking exercise, as demonstrated by the following two quotes that are illustrative of each approach to consent, respectively:

But I think guidelines particularly around where you don’t need to get individual consent. I mean there might be situations where you can get group consent which is something I am very, very comfortable with particularly in anthropology, or you know or get a gatekeeper’s consent. (REC 9)

And, in contrasting a more rigorous approach to consent with a less rigorous tick box for opting out of the research; . . . the question is how in some cases it’s going to be impossible to get anything like traditional voluntary informed consent in which case you’ll have to forgo the benefits that the research data could have accrued or you find some sort of quasi consent process, you know, involving some kind of dodgy opt out type, small print type thing which solves the conscience of the researcher but isn’t consent in any kind of substantive sense. (REC 14)

More experienced REC members also explored the potential for terms and conditions disclosures to operate on a more meaningful level when combined with other safeguards such as anonymization. Such ethical strategies were described as representing a “belt and braces approach” (REC 19) that could be used to further minimize risk to participants:

. . . we took an ethical stance on the project that I’m talking about by using discussion forums from organizations where they make it clear in the terms and conditions that data is publicly available, you don’t have to register to read it. And also that the research might or the data may be used by third-parties for research purposes. So, we took a decision to go with that and so, that combined with the anonymisation is almost like a belt and braces approach. So, we’ve done everything we can to mitigate the risks of harm coming to individuals but we can’t stop it completely.

Relatedly, more experienced REC members emphasized that terms and conditions consent within SM platforms need to find ways to “make it clear . . . that data is [to be considered as] publicly available” (REC 19) so as to avoid privacy violations through what has been regarded as “improper access” by some authors exploring SM research (Zimmer, 2010). Best practice safeguards represent how REC members negotiated consent in relation to the idiosyncratic nature of SM data and the practical difficulties in obtaining permission from SM users. Far from being dismissive of the ethical complexities when reviewing SM research, these interviewees were just keener to engage with new ideas and ethical approaches to help facilitate a research project being conducted ethically without the necessary burden of obtaining traditional consent:

I think we need to be creative . . . I mean for any type of research we see that consent has a very clear historical route . . . but now it just doesn’t serve the purpose . . . I wonder whether we should in some cases get rid of the informed consent altogether. (REC 18)

Indeed, as REC 8 stated, the discussion should perhaps be less about “whether or not you should be using public domain information” and more about “how you actually handle that kind of information, that kind of knowledge in a way that is responsible”—a point echoed by more experienced REC members and best practice proponents (Vayena et al., 2016) alike. And while one experienced REC interviewee took a particularly principled stance in relation to consent where the “right not to be researched and the right not to be observed” (REC 13) was emphasized, this position did not preclude research in the absence of consent for this REC member, but rather necessitated the need for a contextualized, process-based and “very refined ethical strategies and ethical guidance which lets people know how and why you might use those data” (REC 13). This emphasizes the need for more participatory practices which this interviewee noted should ideally reflect more sustained versions of consent rather than research practices where ethics and consent are seen as a “one-off” (Hutton & Henderson, 2015):

And I think there’s an additional step here, which is ethics are seen to be a one-off process . . . I don’t think that we consider enough the role of participants in informing the development of ethical strategies . . . Involve your participants, ask the people whose data you were using . . . ethics are practices of power, I think they’re relational, I think they are things that we do with others and they shape the relationships that we have with them. (REC 13)

Overall then, for experienced REC members consent was not a compulsory necessity of good ethical practice, but rather the decision to require consent was balanced with the perceived risk and harm to SM users produced by the idiosyncratic qualities of each study.

## Discussion

This study demonstrates that REC members hold broad conceptualizations of risk and consent in SM research, and it goes on to empirically study how these conceptualizations are brought into the ethical decision-making process via the views of individual REC members with differing and varied opinions of how consent and risk are to be applied in the SM context (Samuel, 2017). While REC members struggle to reconcile the competing arguments that are presented by SM, this study shows that they are aware of them, and they actively incorporate them into their ethical decision making. It has also explored how these views are related to the REC members’ level of experience, showing discernible trends that were related to REC members’ professional experience of SM research and/or review.

Whereas differences in views between REC members are an expected part of group dynamics, and are a strength of academic groups assessing ambiguous or untested criteria (Derrick, 2018), for SM these views are further complicated by the differing understandings of whether virtual data are considered public or private. In this study, the level of REC member experience of reviewing and also conducting SM research shaped their perception of risk and the necessity of applying different levels of consent (traditional or negotiated). REC members with less experience of SM research and review had polarized views in terms of when consent was or was not required, supporting Vayena et al.’s (2016) observation that perceptions of privacy are often “based on a strict binary conception of identifiability or public availability” (Vayena et al., 2016, p. 435). In this conception, consent becomes the go-to standard of ethical accountability if either identifiability or public availability is in any doubt. Correspondingly, de-identification becomes the go-to ethical strategy if REC members doubted the extent to which data were publicly available or the necessary level of consent was impractical or not possible. For Vayena et al. (2016), binary conceptions of identifiability or public availability, as discussed above, are inadequate for SM research because de-identification can often represent an insufficient protection in the presence of technological advances that make re-identification a relatively simple task. As such, these authors suggest that users are often inadequately protected when de-identification is the go-to standard of protection and also when an approach “based solely on notice and consent where subjects have “limited opportunities to withhold, revoke, or modify consent” (Vayena et al., 2016, p. 431) is taken.

In contrast, more experienced REC members were better able to negotiate consent, basing their ethical decision making on more nuanced conceptions of risk in the online context where data were neither “fair game” nor off-limits without consent. This more nuanced approach in relation to risk and consent emphasized responsible usage of SM data. As such, it is in line with the recommendations of Hutton and Henderson (2015) where perceived privacy of SM users can be negotiated via data anonymization and the application of best practice when secured consent (Hutton & Henderson, 2015) is unavailable, either because of the practical difficulties obtaining it or because of the advantages to be gained from forgoing user permission. In addition, some of these REC members highlighted participatory practices and dynamic forms of consent (Hutton & Henderson, 2015) that could respond to the individual context of each project to provide more meaningful, responsive, and democratic approaches to participant permission.

This more nuanced approach to risk and consent is also more in line with Vayena et al.’s (2016) recommendation that we broaden conceptualizations of old ethical norms for SM research and that “researchers and review boards should be encouraged to incorporate systematic risk-benefit assessments [of SM research] and new procedural and technological solutions from the wide range of interventions that are available” (p. 421). However, while more experienced REC members’ approach to ethical decision making was more nuanced, and moving more toward Vayena et al.’s (2016) recommendations, they still tended to fall back on binary notions of identifiability and consent in the context of public un/availability, that is, although they considered publicly available data as being in need of refined ethical strategies to allow its responsible use, they did not always have a clear idea about what these strategies beyond identification and consent may be. Rather, they tended to articulate that consent did not “serve the purpose” in the contemporary context of SM and that a “belt and braces” approach combining ethical safeguards to minimize risk to participants was therefore required. This upholds Vayena et al.’s (2016) call for a new ethical framework based on “a modern understanding of privacy” (p. 435) focusing on the increased potential to learn about others that SM provides and a reduced reliance on the binary requirements of consent and de-identification.

## Best Practices

Participant protections in the context of ostensibly publicly available data are less obvious on both an ethical and practical basis. To avoid privacy violations based on the improper access that has been raised as a concern by some SM researchers (Zimmer, 2010), it is vital that RECs are able to gain a tacit understanding of the complexities of SM research where privacy, user awareness, and consent combine to obfuscate the core ethical principle of “do no harm.” This will involve REC members gaining important experience in SM research and review, so they are better able to align with the modern understanding of privacy and adequate approaches to protecting participants that are emphasized by Vayena et al. (2016) and also Hutton & Henderson (2015). More experienced REC members came close to this ideal, but they still fell short in terms of moving beyond traditional perceptions of de-identification and consent as representing adequate safeguards to mitigate risk—particularly in the context of “low-risk” aggregated data. Creating sustainability and establishing trust (Vayena et al., 2016, p. 435) in research that includes an SM component requires a broad toolkit of protections that acknowledge ethics as practices of power and take into account the unique challenges to privacy that SM research creates. Such protections should focus less on the binary constructs of consent and de-identification, and more on the qualitative nature of risk within individual studies, to protect participants and to facilitate research that makes good use of the opportunities that SM affords.

## Research Agenda

Challenges to the ethical conduct of SM research will continue to evolve rapidly as technology moves forward and as privacy concerns and research regulatory structures change on a global basis. Research should continue to monitor and evaluate risks within the online context, and to seek procedural solutions to the protection and confidentiality of data, respect for the individual, and the personal privacy of those whose data are captured for research purposes. This research should also explore different, innovative types of consent which might be more relevant for SM users and researchers who wish to use their data.

## Educational Implications

These results have implications for all parties involved in research that utilizes SM data including RECs, researchers, research supervisors, and research subjects. In particular, providing REC members who may be unfamiliar with SM on a personal or professional basis with a tacit understanding of consent, risk, and participant protections in the online context would be a useful way to reduce the opacities associated with the ethics of SM research.

## Notes

1. U.K. Research Excellence Framework, 2014 http://www.ref.ac.uk/

2. Here we talk about do no harm and consent/identification—but there was also a lot of ethical discussions about the legalities, data storage, and so forth, that are not discussed here.

3. It is worth noting that not all SM research goes through ethical review due to perceptions of SM research as a low-risk context where the notion of harm is mitigated by the lack of physical interaction.
